# Comparative evaluation of clinical and radiographic success for Biodentine and MTA in primary molar pulpotomy

**DOI:** 10.6026/973206300214855

**Published:** 2025-12-15

**Authors:** Pinky Chaurasia, Juhi Notra, Nikita Turkar, Sujo Mathew, Chinmayee Dighraskar, Mohamed Tharwat Salama

**Affiliations:** 1 Department of Pediatric and Preventive Dentistry, Triveni Institute of Dental Science and Research Center, Bodri Bilaspur, Chhattisgarh, India; 2 Department of Pediatric and Preventive Dentistry, New Horizon Dental College and Research Institute, Sakri, Bilaspur, Chhattisgarh, India; 3 Department of Pediatric and Preventive Dentistry, Senior lecturer Maitri college of dentistry and research centre, Anjora, Durg, Chhattisgarh, India; 4 Department of Pedodontics, Travancore Dental College, Kollam, Kerala, India; 5 Department of Pediatric and Preventive Dentistry, New Horizon Dental College and Research Institute, Sakri, Bilaspur,Chhattisgarh, India; 6 Department of Orthodontics and Pediatric Dentistry Department, College of Dentistry, Qassim University, Saudi Arabia

**Keywords:** Biodentine, Mineral Trioxide Aggregate, pulpotomy, primary molars, pediatric dentistry, bioactive materials, randomized clinical trial

## Abstract

Vital pulpotomy is essential for preserving primary molars and while MTA is the gold standard, Biodentine has emerged as a promising alternative. Therefore, it is of interest to compare Biodentine and MTA in 120 primary molars over a 12-month follow-up. Biodentine showed a 93.3% success rate versus 91.7% for MTA, with no significant differences in clinical or radiographic outcomes. Procedural time was significantly shorter with Biodentine and both materials showed similarly low rates of internal resorption and furcation radiolucency. Thus, biodentine provides comparable success to MTA with added efficiency, supporting its use as a reliable pulpotomy material in primary molars.

## Background:

Dental caries remains the most prevalent chronic disease affecting children worldwide, with primary molars being particularly susceptible due to their morphological characteristics and eruption timing [1]. When carious lesions progress to involve the pulp, preservation of pulp vitality through vital pulpotomy procedures represents the treatment of choice, maintaining tooth structure and function until physiological exfoliation [2]. Successful pulpotomy depends critically on the pulpotomy medicament's ability to maintain residual pulp vitality, promote healing and provide an effective seal against bacterial microleakage [3]. Historically, formocresol served as the gold standard pulpotomy agent for decades, though concerns regarding its mutagenic and carcinogenic potential have prompted a paradigm shift toward biocompatible alternatives [4]. Ferric sulfate and glutaraldehyde were subsequently introduced, yet concerns about fixation-induced inflammation and incomplete pulpal healing persisted [5]. The development of bioactive calcium silicate-based materials revolutionized vital pulp therapy by offering superior biocompatibility, dentinogenic properties and favorable sealing ability [6]. Mineral Trioxide Aggregate (MTA), introduced in the 1990s, has emerged as the contemporary gold standard for vital pulp therapy in both permanent and primary teeth [7].

MTA's success stems from its excellent biocompatibility, antimicrobial properties, ability to stimulate hard tissue formation and superior sealing ability in the presence of moisture [8]. Clinical and radiographic success rates exceeding 90% at 24-month follow-up have been consistently reported for MTA pulpotomies in primary molars [9]. However, MTA presents certain limitations including prolonged setting time (approximately 2.5-4 hours), difficult handling characteristics, potential for tooth discoloration, high material cost and technique-sensitive manipulation [10]. Biodentine (Septodont, Saint-Maur-des-Fossés, France), a recently introduced calcium silicate-based bioactive material, was specifically designed to address MTA's clinical limitations while maintaining comparable biological properties [11]. Biodentine's composition includes tricalcium silicate, calcium carbonate, zirconium oxide as radiopacifier and a water-based liquid containing calcium chloride as setting accelerator and modified polycarboxylate as water-reducing agent [12]. Key advantages include rapid setting time (10-12 minutes), superior mechanical properties approaching dentin, simplified handling in capsulated form and absence of discoloration [13]. Preliminary in vitro and short-term clinical investigations have demonstrated Biodentine's promising biocompatibility, dentinogenic potential and antimicrobial properties comparable to MTA [14]. However, long-term clinical evidence comparing Biodentine and MTA specifically in primary molar pulpotomy remains limited, with most existing studies reporting follow-up periods of 6 months or less. Furthermore, previous investigations have shown inconsistent results, with some reporting superior outcomes for Biodentine while others found comparable or inferior performance [15]. Therefore, it is of interest to comprehensively compare the clinical and radiographic success of Biodentine versus MTA as pulpotomy agents in primary molars over a 12-month follow-up period.

## Materials and Methods:

## Study design and ethical approval:

This prospective, randomized, parallel-group clinical trial was conducted at the Department of Pediatric and Preventive Dentistry, University Dental Hospital, between January 2022 and March 2023. The study protocol was approved by the Institutional Ethics Committee (approval number: UDH/IEC/2021/234) and registered with the Clinical Trials Registry of India (CTRI/2022/01/039847). Written informed consent was obtained from parents/guardians and verbal assent was obtained from participating children.

## Sample size calculation:

Sample size was calculated based on previous literature reporting MTA success rates of approximately 90% at 12 months. Assuming non-inferiority margin of 10%, α=0.05, power=0.80 and accounting for 20% attrition, a minimum of 54 teeth per group was required. To enable robust subgroup analysis, 60 teeth per group (total n=120) were included.

## Participant selection:

## Inclusion criteria:

[1] Children aged 4-8 years

[2] Cooperative behavior (Frankl score 3 or 4)

[3] Primary molars with deep carious lesions involving pulp

[4] Restorable tooth structure

[5] Absence of clinical signs of irreversible pulpitis (spontaneous pain, nocturnal pain, pain on percussion)

[6] Absence of radiographic evidence of pathology (furcation/periapical radiolucency, internal/external resorption, pathological root resorption >2/3)

[7] At least 2/3 root length remaining

[8] Pulpal hemorrhage controlled within 5 minutes of amputation

## Exclusion criteria:

[1] Medically compromised children with systemic diseases affecting healing

[2] Children with bleeding disorders or immunodeficiency

[3] Uncooperative children requiring sedation/general anesthesia

[4] Teeth with spontaneous pain, swelling, sinus tract, or pathological mobility

[5] Radiographic evidence of internal resorption, furcation involvement, or periapical pathology

[6] Non-restorable teeth

[7] Previous pulp therapy on the tooth

[8] Excessive hemorrhage after pulp amputation (>5 minutes

## Randomization and allocation concealment:

Eligible teeth were randomly allocated to treatment groups using computer-generated random numbers in permuted blocks of 10 (to ensure balance). Allocation sequence was concealed in sequentially numbered, opaque, sealed envelopes opened immediately before pulpotomy procedure by a dental assistant not involved in treatment or assessment. Due to distinct material appearances, operator blinding was not feasible; however, outcome assessors remained blinded to group allocation throughout the study.

## Intervention protocol:

All procedures were performed by two calibrated pediatric dentists (>5 years experience, inter-examiner agreement κ=0.89) under standardized protocol:

## Pre-treatment:

[1] Radiographic examination (intraoral periapical radiograph, bisecting angle technique)

[2] Local anesthesia (2% lidocaine with 1:200,000 epinephrine)

[3] Rubber dam isolation

[4] Caries excavation using slow-speed round burs with copious water spray

[5] Deroofing of pulp chamber with high-speed sterile round diamond bur

## Pulpotomy procedure:

[1] Coronal pulp amputation using sharp sterile excavator

[2] Hemostasis achieved using sterile cotton pellets moistened with saline, applied with gentle pressure

[3] If hemostasis not achieved within 5 minutes, case excluded and recorded

[4] Pulp chamber irrigation with sterile saline

[5] Gentle drying with sterile cotton pellets (avoiding desiccation)

## Group 1 - Biodentine:

[1] Biodentine (Septodont) prepared according to manufacturer's instructions: 1 capsule triturated for 30 seconds at 4,000 rpm

[2] Material applied to pulp chamber floor and radicular pulp stumps using amalgam carrier and condenser, thickness approximately 2-3mm

[3] Setting allowed for 12 minutes

## Group 2 - MTA:

[1] White ProRoot MTA (Dentsply Tulsa Dental) mixed with sterile water at powder:liquid ratio 3:1 to achieve putty-like consistency

[2] Material placed on pulp chamber floor and radicular pulp stumps, thickness approximately 2-3mm

[3] Moist cotton pellet placed, temporary restoration (IRM, Dentsply) applied

[4] Patient recalled after 24 hours for permanent restoration placement

## Final restoration:

[1] For Biodentine: immediate placement of resin-modified glass ionomer (GC Fuji II LC, GC Corporation) followed by stainless steel crown cementation

[2] For MTA: after 24 hours, temporary restoration removed, GC Fuji II LC placed, followed by stainless steel crown

## Procedural time:

Recorded from pulp exposure to final material placement (excluding crown cementation)

## Follow-up and outcome assessment:

## Clinical evaluation:

Performed at 1, 3, 6, 9 and 12 months by blinded examiners (two independent pediatric dentists, inter-examiner agreement κ=0.92).

## Assessment included:

[1] Spontaneous pain (present/absent)

[2] Pain on percussion (present/absent)

[3] Soft tissue swelling (present/absent)

[4] Sinus tract formation (present/absent)

[5] Pathological mobility (present/absent, >Grade 2)

## Radiographic evaluation:

Periapical radiographs obtained at 6 and 12 months using standardized technique (bisecting angle, XCP positioning device). Independent evaluation by two blinded pediatric dentists and one oral radiologist (inter-examiner agreement κ=0.87).

## Assessment included:

[1] Internal resorption (present/absent)

[2] External resorption (present/absent)

[3] Furcation radiolucency (present/absent)

[4] Periapical radiolucency (present/absent)

[5] Widening of periodontal ligament space (present/absent)

[6] Pathological root resorption (>2/3 root length)

## Success criteria:

[1] Clinical success: Absence of all clinical signs/symptoms listed above

[2] Radiographic success: Absence of all radiographic pathologies listed above

[3] Overall success: Both clinical AND radiographic success

## Statistical analysis:

Data were analyzed using SPSS version 26.0 (IBM Corp., Armonk, NY, USA). Descriptive statistics included frequencies and percentages for categorical variables, mean ± standard deviation for continuous variables. Normal distribution was assessed using Shapiro-Wilk test. Chi-square test compared categorical outcomes between groups; Fisher's exact test was used when expected cell frequencies <5. Independent t-test compared continuous variables (procedural time). Kaplan-Meier survival analysis evaluated cumulative success over time, with log-rank test comparing survival curves. Relative risk with 95% confidence intervals was calculated for failure outcomes. Inter-examiner reliability was assessed using Cohen's kappa coefficient. Statistical significance was set at p<0.05 (two-tailed). Per-protocol analysis was performed; sensitivity analysis included intention-to-treat approach considering dropouts as failures.

## Results:

Of 178 children screened, 80 children with 120 eligible teeth were enrolled and randomized (60 teeth per group). Ten teeth (8.3%) were lost to follow-up: 4 in Biodentine group (6.7%) and 6 in MTA group (10.0%). Reasons included: family relocation (n=6), failure to attend appointments despite repeated reminders (n=3) and voluntary withdrawal (n=1). Complete 12-month data were available for 110 teeth (91.7%): 56 Biodentine, 54 MTA. Baseline characteristics were comparable between groups: mean age (Biodentine: 6.2 ± 1.3 years, MTA: 6.4 ± 1.2 years, p=0.423), gender distribution (Biodentine: 53.6% male, MTA: 55.6% male, p=0.830), tooth type distribution (p=0.687) and arch location (p=0.745). Table 1 (see PDF) presents cumulative success rates at different follow-up intervals. Table 2 (see PDF) presents detailed clinical and radiographic outcomes at 12-month follow-up. Table 3 (see PDF) presents procedural parameters and timing data. Mean procedural time was significantly shorter for Biodentine (12.4 ± 2.3 minutes) compared to MTA (15.8 ± 2.7 minutes), representing 21.5% time reduction (p<0.001). Operators rated Biodentine handling as easier (90.0% vs 63.3% rated "easy", p<0.001). Biodentine enabled single-visit completion with immediate final restoration, while MTA required two appointments. Among 9 total failures (4 Biodentine, 5 MTA), timing distribution was: 6 months (n=5, 55.6%), 9 months (n=1, 11.1%) and 12 months (n=3, 33.3%). Biodentine failures included: internal resorption (n=2), furcation radiolucency with mild pain on percussion (n=1) and periapical radiolucency (n=1). MTA failures included: internal resorption (n=2), external resorption with pathological mobility (n=1), periapical radiolucency with pain (n=1) and furcation involvement (n=1). No demographic or clinical factors (age, gender, tooth type, arch) were significantly associated with failure in either group (all p>0.05).

## Discussion:

This 12-month randomized clinical trial demonstrates that Biodentine achieves clinical and radiographic success rates (92.9%) comparable to MTA (90.7%) as a pulpotomy agent in primary molars, while offering significant advantages in procedural efficiency, handling characteristics and treatment completion in a single visit. These findings support Biodentine as a viable alternative to MTA for vital pulp therapy in primary teeth [1]. The overall success rates observed in both groups (>90% at 12 months) align with previous systematic reviews reporting MTA success rates of 88-97% in primary molar pulpotomy [2]. Our findings corroborate recent investigations demonstrating non-inferior performance of Biodentine compared to MTA, extending the evidence base to 12-month follow-up [3]. The comparable success rates reflect similar fundamental properties of these calcium silicate-based materials, including biocompatibility, ability to stimulate reparative dentin formation, alkaline pH providing antimicrobial effects and excellent sealing ability [4]. The mechanisms underlying successful vital pulpotomy with calcium silicate materials involve multiple biological processes [5]. Both Biodentine and MTA release calcium and hydroxyl ions, creating an alkaline environment (pH >12) that is bactericidal and promotes hard tissue formation. The released calcium ions interact with tissue phosphates to form hydroxyapatite, providing a mineralized scaffold for reparative dentinogenesis [6]. Additionally, these materials demonstrate excellent biocompatibility with minimal cytotoxicity, supporting cellular proliferation and differentiation of odontoblast-like cells [7]. The absence of significant differences in specific clinical outcomes (pain, swelling, pathological mobility) between groups suggests equivalent biological responses and healing patterns [8]. The low incidence of postoperative pain in both groups (<4%) reflects successful maintenance of pulp vitality and absence of inflammatory complications. This contrasts favorably with formocresol pulpotomy, which has been associated with chronic inflammation despite clinical success [9]. Internal resorption, the most common radiographic finding in this study (Biodentine: 3.6%, MTA: 5.6%), represents a recognized complication of vital pulp therapy related to chronic inflammation of residual pulp tissue [10]. The comparable incidence between groups suggests similar inflammatory responses. While internal resorption technically represents treatment failure, its slow progression in primary teeth approaching exfoliation may not require intervention unless symptomatic or affecting permanent successor development [11]. The significantly reduced procedural time with Biodentine (12.4 vs 15.8 minutes, 21.5% reduction, p<0.001) represents an important clinical advantage, particularly in pediatric populations where minimizing treatment duration enhances cooperation and reduces anxiety [12]. This efficiency stems from Biodentine's rapid setting time (12 minutes vs >2 hours for MTA), eliminating the need for two-visit protocols and temporary restorations. Single-visit completion reduces appointment burden for families, decreases risk of temporary restoration failure and may improve overall treatment acceptance [13]. The superior handling characteristics reported for Biodentine (90% rated "easy" vs 63% for MTA) reflect its pre-capsulated formulation ensuring consistent powder:liquid ratio and eliminating mixing variability. The homogeneous, putty-like consistency facilitates placement and adaptation to pulp chamber floor [14]. MTA's granular consistency and sensitivity to water ratio can result in washing out with moisture contamination or difficulty achieving proper condensation [15]. The absence of tooth discoloration, though not formally assessed in this study, represents another potential advantage of Biodentine over gray MTA. While white MTA formulations address this concern, Biodentine's zirconium oxide radiopacifier eliminates bismuth oxide present in MTA, which has been implicated in grayish discoloration through interaction with tissue components. Several study strengths warrant acknowledgment. The randomized design with adequate sample size, blinded outcome assessment, standardized treatment protocols, multiple experienced operators ensuring generalizability and comprehensive evaluation at multiple time points enhance validity and reliability. The 8.3% loss to follow-up rate is acceptable for clinical trials and sensitivity analysis treating dropouts as failures did not alter conclusions. Important limitations include the 12-month follow-up duration, which, while adequate for assessing early success, is insufficient for evaluating long-term outcomes until physiological exfoliation. Primary molars remain in function for several years and late failures may occur. Second, radiographic assessment employed conventional periapical radiography rather than cone-beam CT, potentially missing subtle three-dimensional changes. Third, histological assessment of pulpal responses was not feasible in this clinical study, limiting understanding of biological healing mechanisms. Fourth, the study was conducted at a single academic center, potentially limiting generalizability to diverse practice settings. Fifth, cost-effectiveness analysis was not performed, though material costs favor MTA in many markets. Future research should address these limitations through longer-term follow-up studies (24-36 months or until exfoliation), multi-center trials encompassing diverse populations and practice settings, histological studies in animal models examining pulpal healing responses, investigation of Biodentine performance in teeth with varying degrees of pulpal inflammation and cost-effectiveness analyses from healthcare system and patient perspectives.

## Conclusion:

Biodentine demonstrated clinical and radiographic success comparable to Mineral Trioxide Aggregate in primary molar pulpotomy at 12 months, with no significant differences in treatment outcomes. Its superior handling properties, reduced procedural time, and single-visit applicability offer clear practical advantages in pediatric dental practice. Biodentine can therefore be considered an effective and efficient alternative to MTA for vital pulp therapy in primary molars.

## References

[1] Eshghi A (2022). Int J Dent..

[2] Singla R (2023). J Conserv Dent Endod..

[3] Lepcha J (2025). J Indian Soc Pedod Prev Dent..

[4] Jassal A (2023). Int Endod J..

[5] Abuelniel GM (2020). Dent Traumatol..

[6] Baranwal HC (2022). J Conserv Dent..

[7] Tzanetakis GN (2023). Int Endod J..

[8] Anta S (2022). J Endod..

[9] Uyar DS, Alacam A (2021). Niger J Clin Pract..

[10] Abuelniel GM (2021). Eur J Paediatr Dent..

[11] Alyahya A, Qudeimat MA (2024). J Dent..

[12] Ramanandvignesh P (2020). Laser Ther..

[13] Tungjitphianpong P (2024). Int J Paediatr Dent..

[14] Ahuja S (2020). Int J Clin Pediatr Dent..

[15] Alnassar I (2023). Clin Exp Dent Res..

